# A Systematic Review of the Factors Associated with Performance in Non-Elite Runners

**DOI:** 10.3390/jfmk11010124

**Published:** 2026-03-18

**Authors:** Mabliny Thuany, Mayara Silva, Matheus Fernandes, Beat Knechtle, Katja Weiss, Thomas Rosemann, Thayse Natacha Gomes, Ramiro Rolim, Marcos André Moura dos Santos

**Affiliations:** 1 Sports Department, State University of Pará, Tucuruí 68464-000, Pará, Brazil; mablinysantos@gmail.com; 2 Centre of Research, Education, Innovation and Intervention in Sport (CIFI2D), Faculty of Sport, University of Porto, 4200-450 Porto, Portugal; coachmayarasilva@gmail.com (M.S.); rrolim@fade.up.pt (R.R.); 3 Keizo Asami Institute of Immunopathology, Federal University of Pernambuco, Recife 50670-901, Pernambuco, Brazil; matheus.sfernandes@ufpe.br; 4 Medbase St. Gallen Am Vadianplatz, 9000 St. Gallen, Switzerland; katja@weiss.co.com (K.W.); thomas.rosemann@usz.ch (T.R.); 5 Institute of Primary Care, University of Zurich, 8091 Zurich, Switzerland; 6 Department of Physical Education, Federal University of Sergipe, São Cristóvão 49100-000, Sergipe, Brazil; thayse_natacha@hotmail.com; 7 Department of Physical Education and Sports Science, Physical Activity for Health Research Cluster, Health Research Institute, University of Limerick, V94 T9PX Limerick, Ireland; 8 Associated Postgraduate Program in Physical Education, University of Pernambuco and Federal University of Paraiba, Recife 50100-010, Pernambuco, Brazil; mmoura23@gmail.com

**Keywords:** endurance, performance, exercise, non-elite runners, systematic review

## Abstract

**Background**: We aimed to (i) identify the factors associated with performance in non-elite runners, (ii) present the terms and definitions/attributes used to characterize runners, and (iii) identify how performance has been operationalized. **Methods**: Our search was conducted using the databases PubMed, Web of Science, Medline Ovid, Cochrane, PsycInfo, Scielo, Scopus, and SportDiscus in October 2023 and updated in February 2026. Original articles that assessed factors associated with performance in non-elite runners competing in distances ranging from 5 km to ultramarathons were included. The findings were summarized by race distance. The Joanna Briggs Institute Analytical Cross-Sectional Studies critical appraisal tool was used for quality assessment. **Results**: A total of 4151 studies were identified, and 66 studies were included in the final selection. “Recreational” and “athletes” were the most used terms, and finish time was the most common indicator of performance. Performance decline was influenced by arm circumference and mid-axillary skinfold thickness, smoking, body mass index, alcohol consumption, and weather characteristics. Training variables, physiological determinants, and social variables were positively related to performance. **Conclusions**: The field struggles with a lack of clarity regarding the nomenclature and criteria used to categorize runners. The relevance of a predictor differs according to race distance, with physiological aspects becoming less important at higher distances (i.e., marathon and ultramarathon).

## 1. Introduction

Performance in long-distance running is a multifactorial phenomenon, influenced by a plethora of factors related to the subject and the environment [1]. In general, anthropometric, training, and physiological variables have been considered to be important predictors of performance for runners at different competitive levels [1]. For example, a negative association between body fat, body mass, body mass index, and performance has been shown, while training and physiological determinants have shown a positive association [2,3]. Additionally, physiological determinants and training experience have been found to influence marathoners [2,4], with increased maximal oxygen uptake, anaerobic threshold, improved running economy, weekly training volume, and intensity leading to reduced time [2].

Beyond these domains, psychological variables and social support have been investigated. In ultra-trail runners, performance has been associated with mental toughness, resilience, and obsessive passion [5]. While no significant influence has been observed for social support among Brazilian amateur runners competing in 5 km, half-marathon, and marathon events [6], French track-and-field athletes have been shown to be positively influenced by family members [7]. Additionally, environmental factors such as wind, temperature, and humidity [8], as well as race course characteristics, exert an influence on athletes competing in marathon and ultramarathon events [9]. These results indicate the need for a context-sensitive approach.

Despite the multitude of studies aimed at addressing the gap regarding factors associated with performance, the field still grapples with important limitations. A primary limitation pertains to the lack of clarity surrounding the concept of performance [10], including the meaning of performance for non-elite runners, and how performance has been operationalized. A previous study identified that the social representation of performance for amateur athletes includes terms such as “effort”, “improvement”, and “results”, suggesting that performance is perceived as a self-driven objective [11]. Moreover, a wide array of terms has been employed to classify runners. “Amateur”, “recreational”, “well-trained”, “health”, “competitive”, and “novice” are often used interchangeably to describe runners, without clear conceptual distinctions or criteria for differentiation [12].

The heterogeneity of concepts, the diverse training backgrounds of various types of runners, the diverse motivations for engagement in training and competition, and differences in anthropometric characteristics limit guidance, synthesis of the literature, and professional intervention, especially for those focused on performance enhancement [12,13]. Similarly, inconsistency in the application of diverse metrics to evaluate performance in running studies hampers a comprehensive understanding of the subject, which could otherwise offer insights into factors that may be modifiable to enhance outcomes such as health and injury prevention, in addition to performance alone. Gaining clarity on these gaps is the first step to designing interventions to improve running performance while respecting the characteristics and needs of runners.

Given how widespread running is among the general population [14], and given the increasing popularity of ultramarathons in recent decades [15], we aim to identify the factors associated with performance in non-elite runners competing in distances ranging from 5 km to ultramarathons. We hope to advance a previous narrative review that identified predictive models for runners of long-distance races (i.e., 5 km to marathon) at different competitive levels [1] by analyzing the terms, concepts, and attributes used to characterize runners and identifying how performance has been operationalized in running studies.

## 2. Materials and Methods

### 2.1. Protocol and Registration

This systematic review was conducted according to the 2020 Preferred Reporting Items for Systematic Reviews and Meta-Analyses (PRISMA) statement [16] (Supplementary Material File S1) and the PERSiST guidance [17]. Before initiating the review, the protocol for the analysis was registered on the PROSPERO International prospective register for systematic reviews website (https://www.crd.york.ac.uk/PROSPERO/ (accessed on 11 October 2023)) (registration number: CRD42023471483).

### 2.2. Databases, Search Strategy, and Study Eligibility Criteria

A search strategy was developed by the lead author (M.T.) in collaboration with co-authors of this review (T.N.G. and M.S.). The following key databases, relevant to our research question, were searched: PubMed, Web of Science, Medline Ovid, Cochrane, PsycINFO, Scielo, Scopus, and SportDiscus. The search was conducted and completed in October 2023 and updated in February 2026. Table 1 presents the PICOS strategy used to search and the eligibility criteria adopted. We defined non-elite runners as individuals who participate in running without sponsorship and who do not compete in world championships, the Olympic Games, or continental championships. Studies including mixed samples (i.e., both elite and non-elite athletes), as well as those allowing participants to self-classify as elite, were excluded. Articles were excluded if the full text was unavailable or written in a language other than English, Spanish, or Portuguese.

### 2.3. Study Selection

The identification and screening steps were carried out by two authors (M.T. and M.S.). All records retrieved by the search query were imported into the Rayyan tool (https://www.rayyan.ai/ (accessed on 16 December 2023) for systematic review, and duplicates were removed by the lead author (M.T.). Subsequently, the title and abstract were independently screened by the two authors (M.T. and M.S.). Any disagreements were resolved through discussion. Next, the full texts of potentially eligible studies were evaluated independently, and any disagreements were resolved through discussion.

### 2.4. Data Extraction and Research Reporting Quality

Data extraction was performed by the lead author (M.T.), with a quality control cross-check performed by both M.T. and M.F. One week after data extraction, both authors reviewed the extraction of half of the studies. Data extraction included: (1) the first author and year of publication; (2) sample characteristics; (3) term used to classify runners; (4) definition of the term/attributes used to characterize the sample (when reported); (5) performance indicator (e.g., mean speed, finish time, and running pace); (6) variables studied; and (7) main outcomes. The findings were summarized in a general way and by race distance (i.e., 5 km, 10 km, half-marathon, marathon, and ultramarathon).

The Joanna Briggs Institute Analytical Cross-Sectional Studies critical appraisal tool [18] was used for quality assessment. Two reviewers (MT and TNG) conducted individual quality assessments, resolving discrepancies through consensus. The quality appraisal checklist comprised eight criteria: whether (1) the sample inclusions were defined; (2) the study subjects and settings were described; (3) exposure was measured validly and reliably; (4) objective, standard measurement criteria for the conditions were used; (5) confounding factors were identified; (6) strategies to deal with confounding factors were stated; (7) the outcomes were measured validly and reliably; and (8) appropriate statistical analysis was used. Each criterion was scored as being “met = yes” or “not met = no” or “unclear”, or, in some instances, as “not applicable”. To verify the quality of the study, we summed up the “yes” responses. Results were used to assign an a priori quality rating to each study (0–2 points = very low; 3–4 points = low; 5–6 points = moderate; and 7–8 points = high).

## 3. Results

**Figure 1:**
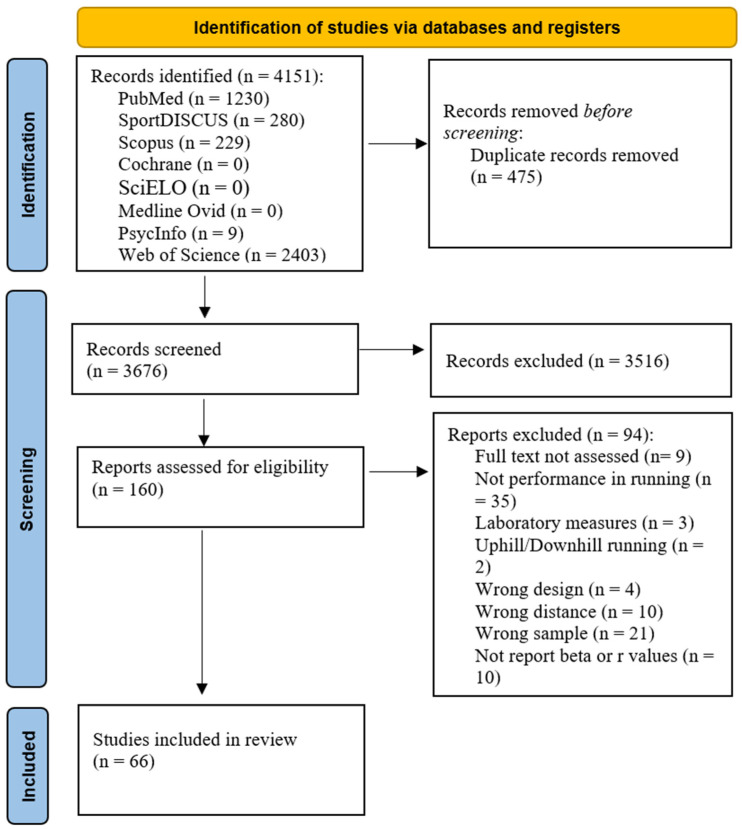
PRISMA flow diagram of the screening process.

The search retrieved 4151 studies. After excluding duplicates, 3676 studies remained. A total of 3516 studies were excluded by title/abstract, and 160 were assessed, of which 94 were excluded. Reasons for exclusion included not assessing performance prediction, laboratory measures, focusing on uphill/downhill running, using the wrong design/distance or sample, and not reporting beta or r values. Upon reading the entire text, 94 studies were excluded, leaving 66 that met the eligibility criteria. Figure 1 shows the search results and screening process.

### 3.1. Research Reporting Quality

Table 2 presents the methodological quality assessment of the included studies. The vast majority of studies (92.1%) were classified as moderate quality, four studies (5.3%) achieved a high-quality rating, and two studies (2.6%) were classified as low quality; no study was rated as very low quality. For question Q1 (“Were the criteria for inclusion in the sample clearly defined?”), approximately 62.1% of the studies were rated as “no”, indicating that clear inclusion criteria were frequently not reported. In contrast, question Q2, which evaluates whether study participants and settings are described in detail, was positively rated for 98.5% of the studies, as most reported participants’ age, sex, training experience, and anthropometric characteristics, although contextual and time frame details were sometimes limited. Regarding questions Q3 and Q4, most of the studies were rated positively, suggesting that exposures and outcomes were generally measured in a valid and reliable manner. For confounding factors (Q5 and Q6), important methodological limitations were observed, with few studies clearly identifying confounding factors (Q5) or reporting strategies to deal with them (Q6), although some applied statistical approaches such as stratification by sex, age, or competitive level. All studies were positively rated for Q7, indicating appropriate validity and reliability of outcome measurements. Similarly, Q8 was rated positively in 100% of the studies, reflecting the use of appropriate statistical analyses aligned with the study objectives, although some studies could have benefited from more detailed reporting.

### 3.2. Sample Characteristics and Terms Used

A total of 2,347,977 runners were sampled from 1988 [63] to 2026 [81]. Of the total, 1,525,555 were men (~65%), and 822,422 were women (~35%). “Recreational” was the term most used (12 studies), followed by “athletes” (11 studies), “well-trained/moderately trained runners” (ten studies), and “runners” (five studies). Less frequent terms were “competitors/competitive”, “endurance runners/endurance trained”, “master”, “trail/ultra-endurance or ultrarunners”, “road runners”, “healthy runners”, “amateur”, “non-professional”, “joggers”, “finishers”, “starting runners”, and “subjects”, and three other studies used two different terms (“recreational” and “nonprofessional athletes”, “recreational healthy runners”, and “endurance-trained”).

Of these studies, only three presented an explanation for the terms used. For example, the term master athletes was used and defined in two studies: Wiswell et al. [51] considered “master athletes” to be runners aged ≥40 years who had at least 5 years of training and a running volume of ≥15 km/week, and who participated at least once per year in organized running competitions. Similarly, Forsyth et al. [72] considered veteran runners to be those aged 40 years or older who had at least five years of training, reached a training volume of at least 32 km/week, and competed at least once per year in organized running competitions. For Scheer et al. [48], trail runners were those who completed the majority of their training on trail surfaces or had previously participated in trail running competitions (Supplementary Table S1).

### 3.3. Race Distance and PerformancePerformance in

The majority of studies focused on ultramarathons [9,20,22,25,29,33,34,35,36,37,41,53,70,73,75,76,78,79,80], followed by 5 km races [19,27,38,44,47,49,57,59,61,66,72,74]. Eight studies investigated half-marathons [4,23,24,26,30,42,54,56,58], while seven studied marathons [21,32,45,60,62,69,81] and 10 km races [39,40,52,65,68,71,77]. Some studies focused on investigating different race distances [6,28,31,43,48,51,55,64,67], with fewer studies investigating distances ranging from 7 miles to 27 km [46,50,63]. Regarding the metrics used to assess performance, the race time/finish time was the most used. Other strategies included the runner’s personal best [47,51,52,57,59], race/average speed [9,43,44,55,75,76,79,80], running pace [6,28,67], meters by seconds [38,49], and the total kilometers covered in time-limited events [25,36].

### 3.4. Factors Associated with Performance

The majority of studies used correlation/regression models to analyze the correlation/association between predictors and performance. The findings are presented according to the correlation coefficients and beta results from the studies, indicating potential performance enhancements or declines depending on the measurement indicator. Because performance was operationalized differently across studies (e.g., race time, HH:MM:SS, running pace, running speed, etc.), the direction of association is interpreted according to the specific outcome reported (Supplementary Table S2).

#### Performance in 5 km Races

Among the 13 studies investigating 5 km races, the majority classified runners as trained (eight studies). The anthropometric variables (e.g., tibia length relative to leg bone length, relative total length of forefoot bones (big toe), relative total length of forefoot bones (second toe), absolute femur and tibia length, absolute total leg bone length of the femur and tibia, femur length relative to leg bone length, femur/tibia ratio of leg bone length, body fat percent, sum skinfold, and fat mass) were generally negatively associated or not significantly associated with performance [19,27,47,57,74]. Similarly, biomechanical variables (passive plantar flexor stiffness, stiffness of tendon structures, maximal voluntary contraction, muscle thickness, maximal elongation of tendon structures, and thickness of tendon) presented a negative or non-significant association with performance [59,66]. BMI, lean mass, and fat-free mass were not associated with race time [74], while body fat percent was positively associated with race time [27,74]. Physiological variables, such as VO_2_max, ventilatory threshold, the velocity at VO_2_max, and energy cost during submaximal running at 14 km, 16 km, and 18 km, showed a positive association [38,44,49,61,72,81], while the study developed by Kilding et al. [44] did not show a significant association between running economy and performance.

### 3.5. Performance in 10 km Races

Of the studies investigating runners competing in 10 km races [39,40,52,65,68,71,77], most primarily measured performance as race time (HH:MM:SS) or personal best. Most of the studies were focused on physiological variables, with only de Anda Martín et al. [77] investigating psychological variables and showing that higher levels of arousal and perceived isolation were positively associated with race time.

Aerobic capacity markers were consistently associated with better performance. Higher VO_2_max and greater power at lactate threshold were positively related to faster race times or superior personal bests [52,71]. Similarly, vVO_2_max was negatively associated with race time, indicating that runners with higher maximal aerobic speed achieved better performance [77]. Peak treadmill speed and respiratory compensation point were also positively associated with performance [71]. In contrast, maximal heart rate during 10 km races and at maximal treadmill speed showed negative associations with race time in one study [40].

Regarding neuromuscular variables, greater maximal dynamic strength, peak velocity in the half squat, peak force in the loaded squat jump, and changes in countermovement jump peak eccentric velocity were associated with better performance [40,71]. Similarly, sprint ability (300 m sprint time) and plyometric leap distance were also related to performance outcomes, both in the total sample and in sex-specific analyses [39]. Training impulse measures were investigated in one study [68]. Individualized TRIMP decreased following both 5 km and 10 km performances. Inflammatory markers were explored by Siqueira et al. [65], who observed that better performance was associated with lower post-race plasma IL-6 levels and higher IL-10 levels, indicating a more favorable anti-inflammatory profile among faster runners.

### 3.6. Performance in Half-Marathon

Race time was used as the outcome in all of the studies that sampled half-marathoners [4,23,24,42,54,56,58]. BMI and the sum of six skinfolds were positively associated with race time [4]. Some studies showed some discrepancy in results regarding the mid-axillary skinfold thickness, body mass, body mass index, body fat percent, and multiple regional skinfolds (pectoral, triceps, subscapular, abdominal, suprailiac, and medial calf) [23,24,26,30]. Increased body weight was linked to poorer treadmill performance and lower vVO_2_max [42], and BMI was positively associated with race time in recreational runners [30].

Higher aerobic capacity indicators were consistently associated with better performance, with a greater VO_2_max and vVO_2_max being related to faster race times [30,42]. Peak speed, respiratory compensation threshold (RCT) speed, RCT speed rate, and maximal step length were negatively associated with race time, indicating that runners with superior physiological efficiency completed the race faster [4]. Performance in the Cooper test (distance covered) was positively associated with race outcomes [42]. Similarly, higher mean training speed and greater weekly training volume were linked to improved performance [30].

Regarding hormonal variables, Radosavljević et al. [54] observed that post-run salivary testosterone concentrations decreased in association with performance, whereas salivary cortisol and pre-run testosterone concentrations showed no significant relationship with race time. Psychological characteristics were examined in one study, showing that higher desired race time reported by runners was positively associated with race time, while higher trait emotional intelligence was negatively associated, indicating that runners with greater emotional intelligence tended to perform better [56].

### 3.7. Performance in Marathon

Among the studies sampling marathoners [21,32,45,60,62,69,81], some were focused on the natural environment, showing that sunshine duration and maximum temperature negatively influenced performance [60]. Training variables positively related to performance included the mean speed of training sessions, years of sports practice, and minutes of daily training, whereas years of athletics practice, days of training per week, and hours of training per week did not show a significant relationship [32,62]. Some physiological variables (e.g., muscle velocity, relative power, and ventilatory threshold) showed no significant association with performance, while mid-axillary skinfold thickness was shown to impair race time [62]. Training characteristics (running more than 10 h/week, more frequent weekly running and quality sessions, greater weekly distance, cross-training sessions, and combined running plus cross-training sessions during the 4–0 months pre-race period) were associated with faster race times, whereas reductions in weekly sessions were linked to slower performance [81].

Neuromuscular, physiological and anthropometric factors demonstrated mixed associations. Higher maximal force (F0), maximal power (Pmax), and ventilatory threshold (Thvent) were positively associated with performance, whereas muscle velocity (v0), relative power (rPmax), and coefficient of variation (CV) showed no significant effect [21,45]. Regarding anthropometry, mid-axillary skinfold thickness was negatively associated with race performance, while other measures, including body mass, BMI, percentage of body fat, and multiple skinfolds (pectoral, triceps, subscapular, suprailiac, abdominal, and medial) did not demonstrate significant associations [62]. In addition, a multivariate model that included 10 km race time, BMI, age, and sex as predictors demonstrated a very high correlation between predicted and actual performance [69].

### 3.8. Performance in Ultramarathon

Running performance in ultramarathons was influenced by a combination of training, demographic, environmental, physiological, and nutritional factors. Predictors of performance included higher total weekly training numbers [78], the personal best marathon, performance in previous marathons [35,36,37], the longest training session before the 24 h race [36], and tactical decisions during the race [29,33]. Additional factors included sex, age [80], coefficient of variation, and sedentary time [41]. Favorable environmental factors, such as running on asphalt or track surfaces and flat elevation, were identified, while wind speed and course characteristics, such as trail, mountain, and hilly terrain, were found to impair performance [9,35,76,79,80]. Nutritional intake also influenced performance, with higher-performance runners presenting increased carbohydrate intake [75]. Additionally, maximal aerobic speed and its fraction [20], maximal power [53], and maximal aerobic power and peak velocity [29] also were associated with a better race time. Several variables showed no significant association with performance, including age, body height and mass, limb circumferences (upper arm, thigh, and calf), skinfold thicknesses (pectoral, axillar, triceps, subscapular, abdominal, suprailiacal, thigh, and calf), the sum of upper- and lower-body skinfolds, BMI, % body fat (%BF), skeletal muscle mass (%SM), years as an active runner, average yearly or weekly training volume, number of completed similar races, resting heart rate, pre- and post-race body temperature, hematocrit values, weight loss, narrow pulse pressure, VO_2_max, VO_2_ and power at VT1 and VT2, mean core temperature, and intermediate physiological variables such as IP [36,53,70,73].

### 3.9. Performance in Different Distance Events

Within the studies that assessed performance at different race distances [6,26,28,31,43,46,48,50,51,55,63,64,67,68], authors used different classifications (i.e., recreational and nonprofessional athletes, road runners, competitive, trail, trained, master, joggers, and endurance-trained) and variables to assess performance (i.e., race time, running pace, speed, and personal best). Beyond anthropometric variables, age, smoking, BMI, socioeconomic level, alcohol consumption, influence to run, perception of the natural environment, and athletics events showed a positive association with performance. Also, physiological and training variables were not associated with performance in runners.

## 4. Discussion

The main purpose of this review was to identify factors associated with performance in non-elite runners. Our secondary aim was to analyze the terms, concepts, and attributes used to characterize runners and to identify how performance has been operationalized in running studies. Given that the number of non-elite runners has increased worldwide [82,83], along with the body of information that can help to understand the running phenomenon, the present systematic review contributes to filling some gaps and advancing the current knowledge. The most valuable information provided by the present review concerns three main factors: (1) the lack of conceptual clarity of terms used in running studies, (2) the metrics used to study performance, and (3) the multifactorial nature of performance.

Regarding the terms used among the studies included in this review, only three studies explained them appropriately. Interestingly, two studies that adopted the terms “master” and “veteran” considered similar criteria in classifying runners [51,72], diverging in the total volume/week adopted (at least 15 and 32 km/week for master and veteran runners, respectively). In addition, one study classified participants based on the geographical characteristics where the race was performed [48]. Furthermore, some studies characterized runners following the recommendations for describing off-road running athletes presented by the Ultra Sports Science Foundation [12]. This information includes demographic data (age, sex, height, and body mass), training characteristics (training load and years of running), and competition characteristics (information about race and competitive level). Despite this, it remains unclear why researchers adopt different nomenclatures (e.g., amateur, recreational, and health, among others). To improve consistency and facilitate comparisons across studies, we propose a minimum reporting set for runners that includes participant demographics, training history, race specialty, recent performance, and eligibility or health criteria (Supplementary Table S3).

Among the included studies, most used the finish time as a performance indicator, which allows comparison between studies. Understanding how performance has been operationalized in running studies poses both theoretical and practical challenges [10]. How running is measured is strongly related to the paradigms adopted by researchers. In this context, performance is considered an output, measured through the final time of the race event, also frequently measured through secondary datasets available on the event’s official webpage. Despite this pragmatic approach offering important practical applications for training, monitoring, and control, time series analysis could provide valuable information on how performance changes according to changes in intrapersonal and environmental factors [10].

Regarding the factors associated with performance, our results highlight the multifactorial nature of performance and reinforce the importance of using a multidisciplinary approach to improve performance while also maintaining health [84]. Several domains were studied, including physiological, anthropometric, training, psychological, biomechanical, and weather characteristics, with the results differing according to the distance. Overall, 5 km and 10 km race performance is predominantly determined by aerobic capacity and neuromuscular performance, similar to a previous study [1]. As race distance increases, training characteristics, experience, environmental exposure, pacing strategy, and nutritional management become progressively more important [9,20,22,25,29,33,34,35,36,37,41,53,70,73,75,76,78,79,80], while traditional laboratory-based physiological markers lose predictive strength. This can be related to the fact that runners competing in marathons or ultramarathons, in particular, need to manage adequate hydration, nutrition, and sleep [73,75,84], which in association with extreme heat, humidity, and wind can increase the challenges and expose runners to maladaptive outcomes and a decline in performance.

The understanding of variables associated with performance has important practical applications for runners, including changes in training schedules to better prepare runners to face challenges in competitions. Managing the plethora of variables associated with performance requires a deep understanding of the basic characteristics of the runners, the distance studied, and integrative work.

### Limitations and Strengths

Firstly, our approach did not allow us to cover all of the scientific literature, especially studies published in languages other than English, Portuguese, or Spanish. Secondly, despite excluding studies sampling elite athletes, we did not control for studies that might have included runners with higher performance levels. The challenge here lies in the absence of a cut-off point to classify athletes as higher level, especially considering the differences in performance across countries, distances, and events. Thirdly, we did not control for the methods used to measure the variables used as independent variables, which can be biased. Fourth, we decided to keep the results as originally presented by the authors, which can create some noise in the understanding of the findings.

Overall, the methodological quality of the included studies was predominantly moderate, with only a small proportion classified as high quality and very few rated as low quality. While most studies provided detailed descriptions of participants and employed valid and reliable measures for exposures and outcomes, important methodological limitations were evident. In particular, clear inclusion criteria were frequently not reported, and confounding factors were rarely identified or adequately addressed. Despite the consistent use of appropriate statistical analyses and reliable outcome measurements, these recurring limitations suggest that the findings should be interpreted with caution, especially regarding potential sources of bias and residual confounding.

Notwithstanding these limitations, the present systematic review presents some advances in the previous literature by summarizing the factors associated with performance among non-elite runners, exploring the terminologies used to classify runners, and examining the performance indicators used. It is beyond the scope of the present review to provide a taxonomy for future studies. Despite this, the classification criteria adopted within the included studies in this systematic review can provide important insights for future investigations, including the need for the establishment of cut-off points.

## 5. Conclusions

Studies assessing predictors of performance in non-elite runners use a range of domains of variables, including physiological, anthropometric, biomechanics, training, psychological, and weather. The relevance of the predictor differs according to the race distance. Among the different terms used, recreational runner was most commonly used by authors, and only three studies provided the criteria used for the adoption and definition of the term adopted. Among the different metrics used to assess performance, race time was the most common.

## Figures and Tables

**Table 1:** Search terms and eligibility criteria following the PICOS strategy.

	Search Terms	Inclusion Criteria	Exclusion Criteria
P	Runners OR “long-distance runners” OR “amateur runners” OR “recreational runners” OR joggers OR “non-professional” NOT “elite-athletes”	Women and men; non-elite runners competing in race distances ranging from 5 km to ultramarathons; runners competing in mountain, trail, or sky runs	Animals and in vitro studies; children or adolescents; elite athletes; triathletes or biathletes;
I	“Factors associated” OR correlates OR predict* OR determinants	Studies reporting predictors, correlations, or associations	Do not focus on running performance or performance predictors
C	Not applied	Not applied	Not applied
O	Performance OR “running pace” OR velocity OR “finish time”	Performance (finish time, velocity, running pace) is the main outcome	Walking; performance in sprint; running performance in team sports; assessing performance in lab tests
S	All designs	Original study articles published in peer-reviewed journals, adopting both quantitative and/or qualitative approaches	Conference abstracts, commentaries, book chapters, or editorials

The asterisk (*) was used as a wildcard to truncate word stems in the search strategy, allowing the retrieval of different variations of the terms sharing the same root.

**Table 2:** Quality assessment for included studies.

Author, Year	Q1	Q2	Q3	Q4	Q5	Q6	Q7	Q8	Total
Ueno, et al. (2021) [19]	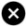	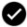	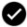	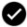	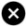	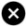	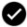	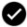	5
Balducci, et al. (2017) [20]	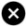	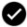	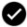	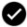	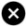	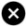	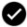	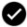	5
Nikolaidis & Knechtle (2020) [21]	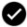	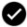	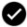	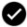	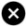	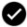	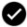	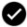	7
Knechtle et al. (2008) [22]	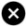	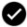	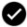	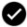	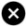	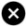	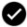	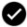	5
Knechtle et al. (2011) [23]	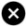	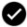	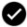	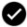	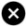	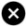	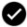	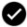	5
Knechtle et al. (2011) [24]	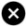	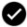	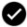	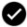	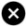	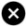	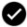	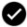	5
Knechtle et al. (2009) [25]	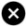	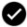	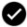	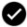	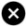	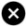	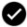	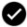	5
Knechtle et al. (2010) [26]	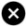	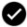	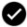	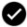	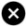	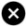	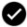	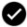	5
Dellagrana et al. (2015) [27]	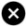	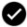	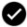	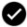	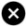	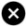	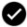	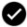	5
Gómez-Molina et al. (2017) [4]	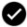	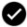	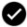	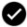	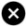	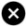	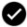	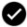	6
Thuany et al. (2023) [6]	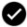	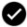	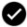	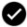	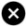	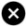	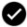	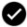	6
Thuany et al. (2021) [28]	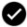	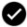	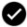	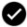	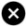	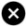	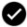	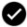	6
Coates et al. (2021) [29]	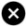	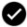	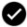	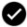	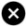	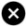	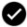	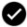	5
Nikolaidis et al. (2023) [30]	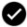	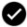	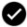	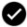	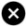	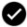	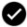	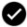	7
Thuany et al. (2021) [31]	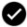	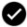	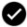	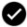	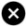	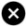	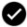	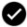	6
Clemente-Suarez & Nikolaidis (2017) [32]	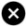	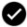	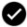	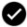	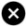	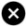	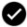	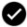	5
Daniela et al. (2012) [33]	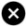	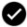	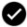	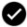	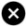	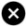	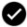	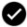	5
Knechtle et al. (2010) [34]	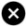	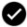	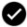	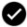	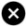	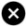	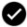	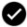	5
Coquart (2023) [35]	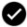	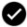	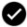	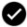	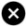	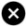	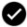	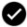	6
Knechtle et al. (2011) [36]	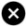	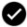	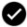	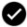	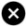	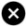	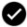	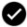	5
Knechtle et al. (2010) [37]	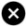	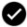	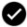	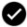	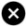	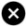	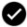	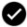	5
Paavolainen et al. (1999) [38]	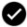	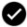	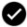	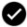	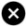	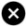	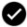	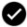	6
Sinnett et al. (2001) [39]	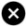	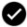	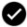	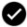	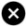	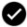	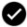	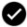	6
Del Rosso et al. (2021) [40]	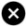	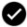	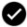	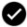	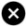	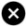	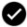	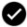	5
Martínez-Navarro et al. (2021) [41]	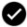	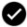	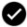	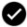	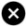	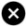	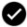	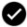	6
Alvero-Cruz et al. (2019) [42]	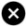	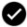	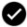	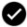	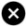	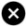	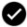	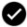	5
Takeshima & Tanaka (1995) [43]	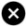	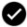	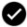	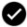	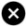	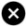	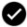	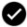	5
Kilding et al. (2006) [44]	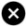	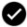	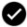	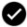	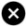	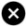	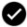	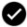	5
Florence & Weir (1997) [45]	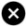	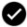	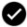	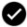	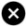	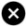	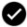	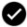	5
Adams et al. (2017) [46]	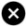	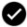	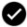	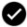	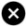	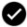	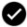	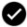	6
Ueno et al. (2019) [47]	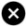	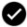	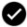	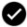	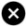	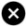	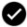	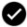	5
Scheer et al. (2019) [48]	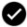	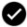	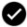	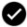	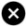	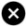	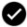	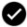	6
Nummela et al. (2006) [49]	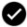	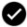	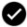	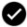	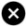	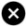	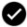	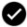	6
Alvero-Cruz et al. (2019) [50]	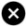	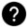	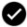	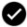	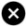	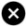	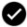	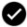	4
Wiswell et al. (2000) [51]	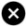	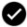	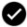	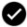	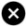	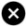	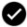	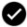	5
Rossuello et al. (2009) [52]	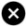	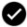	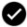	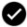	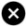	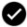	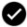	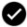	6
Fornasiero et al. (2018) [53]	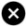	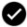	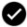	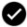	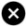	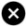	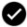	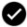	5
Radosavljević et al. (2016) [54]	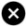	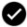	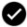	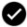	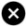	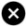	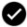	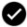	5
Masters & Ogles (1998) [55]	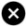	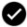	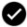	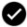	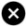	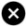	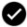	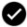	5
Rubaltelli (2018) [56]	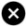	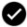	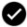	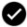	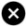	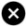	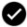	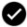	5
Ueno et al. (2018) [57]	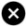	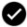	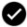	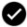	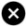	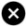	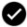	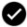	5
Knechtle et al. (2014) [58]	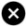	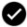	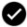	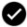	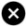	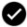	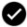	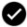	6
Ueno et al. (2018) [59]	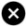	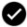	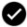	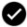	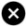	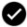	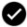	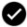	6
Knechtle et al. (2021) [60]	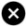	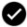	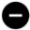	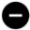	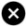	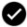	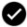	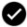	4
Ramsbottom et al. (1989) [61]	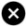	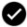	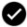	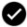	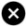	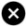	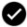	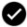	5
Schmid et al. (2012) [62]	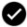	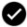	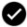	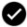	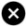	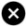	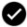	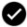	6
Marti et al. (1988) [63]	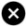	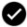	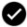	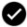	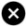	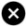	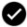	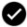	5
Machado et al. (2013) [64]	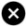	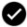	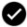	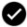	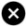	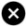	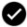	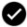	5
Siqueira et al. (2022) [65]	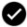	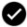	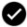	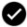	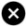	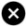	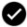	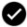	6
Kubo et al. (2015) [66]	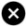	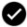	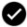	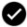	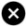	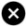	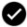	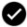	5
Matos et al. (2020) [67]	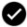	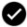	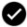	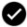	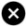	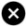	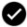	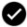	6
Manzi et al. (2009) [68]	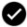	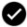	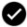	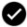	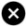	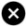	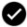	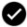	6
Lerebourg et al. (2023) [69]	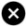	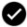	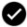	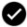	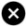	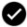	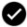	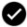	6
Landman et al. (2012) [70]	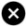	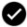	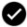	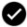	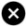	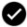	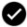	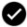	6
Bertuzzi et al. (2014) [71]	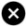	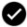	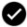	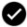	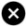	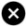	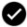	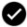	5
Forsyth et al. (2017) [72]	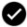	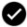	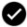	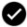	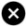	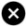	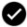	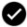	6
Valentino et al. (2016) [73]	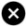	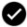	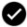	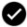	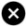	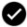	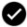	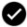	6
Alves et al. (2024) [74]	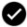	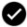	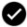	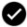	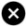	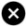	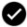	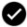	6
Inamura et al. (2024) [75]	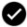	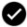	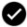	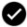	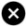	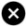	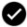	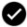	6
Knechtle et al. (2024) [76]	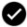	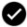	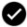	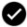	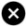	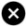	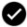	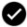	6
de Anda Martín et al. (2024) [77]	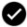	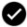	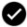	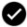	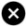	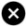	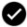	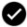	6
Gutiérrez et al. (2025) [78]	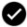	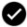	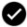	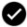	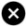	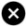	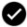	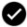	6
Knechtle et al. (2025) [79]	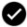	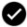	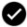	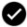	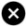	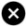	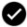	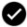	6
Knechtle et al. (2025) [9]	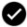	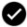	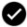	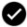	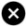	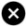	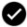	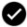	6
Turnwald et al. (2025) [80]	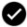	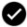	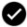	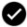	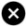	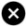	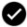	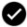	6
DeJong Lempke et al. (2026) [81]	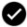	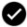	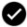	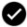	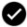	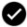	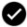	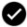	8

Legend: Q1 (Were the criteria for inclusion in the sample clearly defined?); Q2 (Were the study subjects and the setting described in detail?); Q3 (Was the exposure measured in a valid and reliable way?); Q4 (Were objective, standard criteria used for measurement of the condition?); Q5 (Were confounding factors identified?); Q6 (Were strategies to deal with confounding factors stated?); Q7 (Were the outcomes measured in a valid and reliable way?); Q8 (Was appropriate statistical analysis used?). Yes (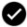); No (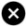); Unclear (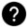); Not applied (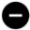). The total is based on the sum of the “yes” responses. The results of this assessment were used to assign an a priori quality rating to each study (0–2 points = very low; 3–4 points = low; 5–6 points = moderate; 7–8 points = high).

## Data Availability

No new data were created or analyzed in this study. Data sharing is not applicable to this article.

## Supplementary Materials

The following supporting information can be downloaded at https://www.mdpi.com/article/10.3390/jfmk11010124/s1: File S1: PRISMA 2020 Checklist; Table S1: The runner’s competitive level of the selected studies and classification criteria used; Table S2: Summary of the main findings; Table S3: Proposed minimum reporting set for runners.
