# Supplementary material for: A Systematic Review of the Factors Associated with Performance in Non-Elite Runners
Source: J Funct Morphol Kinesiol. 2026 Mar 18;11(1):124. doi: 10.3390/jfmk11010124 (PMC13027911; doi:10.3390/jfmk11010124)
Supplement: Supplementary file 1 [file jfmk-11-00124-s001.zip › Supplementary Tables.pdf]

Supplementary Table S1. The runner's competitive level of the selected studies and classification criteria used

Author, year	Term used	Classification criteria
Lempke, A et al 2026	Athletes	Not presented
Gutiérrez, H et al 2025	Trail runners	Not presented
Knechtle, B et al 2025	Athletes	Not presented
Knechtle, B et al 2025	Athletes	Not presented
Turnwald, J et al 2025	Athletes	Not presented
Alves et al, 2024	Men runners	At least one year with practics in 5km
Inamura et al, 2024	Ultramarathoners	Not presented
Knechtle, B et al 2024	Ultramarathoners	Not presented
Martín, I et al 2024	Male runners	Training and competing experience as an athlete (> 5 years practicing this sport), running more than 3 days a week for 45 min, and not being injured at the time of the study.
Thuany et al, 2023	Road runners	Excluded if no information is provided on running pace and preferred distance
Nikolaidis & Knechtle, 2023	Recreational	Successful participation in the Athens Authentic Marathon in 2017; free of injury or illness
Coquart, 2023	Runners	Not presented
Lerebourg et al, 2023	Athletes	Performed the 10-km and the marathon in the same year; athletes who ran a marathon before their 10-km were removed; athletes who maintained a higher speed in the marathon than in the 10-km race were excluded; those with a performance in the 10-km below the lowest ranking of the FFA were eliminated (i.e., performance > 50 and 60 min, respectively, for men and women)
Siqueira et al, 2022	Trained runners	Practicing running for at least 2 years; have participated in two long-distance running races in the last 6 months; properly registered in a 10 km running race
Ueno et al, 2021	Endurance runners	Well-trained; involved in regular training and competition; free of musculoskeletal injury; no self-reported neurological and cardiopulmonary impairments
Thuany et al, 2021	Non-professional runners	Not presented
Coates et al, 2021	Recreational	Healthy; non-smoking individuals
Thuany et al, 2021	Road runners	Not presented

Del Rosso et al, 2021	Well-trained runners	Competing and training for at least 3 years in events; 10 km to half-marathon; minimum 4 training days/week
Martínez-Navarro et al, 2021	Recreational	Previously completed at least one ultramarathon (>60 km)
Knechtle et al, 2021	Finishers	Not presented
Nikolaidis et al, 2020	Recreational	Marathon personal record of 4:02 (0:45 h: min); median 3 completed marathons; performed 4.3 (1.3) running sessions weekly; weekly running distance of 52.7 (21.1 km)
Matos et al, 2020	Recreational	Minimum 600 in the International Trail Running Association performance index; participation in the trail running championship; participation in the national trail running championships; more than three years' experience in the sport; registration in all training sessions and competitions; free of injuries than three consecutive weeks in the 12 months prior
Alvero-Cruz et al 2019	Amateur	Experience in training (8.3 ± 5.65 years) and long-distance races
Ueno et al, 2019	Endurance runners	Well-trained; involved in regular training and competition
Scheer et al, 2019	Trail runners	Not presented
Alvero-Cruz et al, 2019	Trained runners	Not presented
Fornasiero et al, 2018	Recreational healthy runners	No clinical evidence of cardiovascular, neuromuscular, or articular diseases; training experience of 7 (7) years; 3 (3) years of experience in MUMs; about 7 (3) hours/week covering 55 ± 31 km/weekly; intend to complete it in the best time possible
Rubaltelli et al, 2018	Runners	Not presented
Ueno et al, 2018	Well-trained runners	Involved in regular endurance training and competition; personal best 5000-m race times in endurance runners ranged from 834 s to 967 s; mean experience of 7.6 (2.5) years i endurance training.
Knechtle et al, 2018	Competitors	Included only once and recruited continuously during two consecutive years from 2010 to 2011 to increase the sample size
Ueno et al, 2018	Well-trained runners	Involved in regular training and competition; best personal times in a 5000-m race ranged from 858 to 967 s
Balducci et al, 2017	Healthy runners	Healthy; without injuries, not taking any medication
Gómez-Molina et al, 2017	Runners	Phase 1: Completed during the 6 weeks before the study a half-marathon in less than 105 min; Phase 2: runners had to run a half-marathon in the four weeks after testing

Clemente-Suarez & Nikolaidis, 2017	Athletes	Years practicing sports of 12.9 (12.9); years of athletic training of 7.8 (4.7); Mean of 5.2 (2.8) days of training/week; weekly training hours of 10.5 (8.7) hours, and 87.2 (41.9) minutes of daily training
Adams et al, 2017	Not presented	Not presented
Forsyth et al, 2017	Endurance trained, veteran runners	Were ≥35 years, ran ≥15 km per week, were ctively competing at club level, and had been running for at least 5 years.
Radosavljević et al, 2016	Recreational	Physically active throughout the previous year; in good health; drug and injury-free
Valentino et al, 2016	Starting runners	Not presented
Dellagrana et al, 2015	Moderately trained young runners	Minimum of 6 months of experience with training and endurance events; training 6 days per week; weekly volume range of 60 to 80 km
Kubo et al, 2015	Trained runners	Training experience ranged from 2.5 to 14.5 years; participated in competitive meets at the regional or intercollegiate level within the preceding year; the best official record in a 5000 m race within 1 year before these tests ranged from 14:11 to 16:16 min
Bertuzzi et al, 2014	Recreational	Competed regularly in 10-km running races at regional levels; training for the last 2 years without interruption; participated in local competitions; best performances in 10-km competitions ranged from 35 to 45 minutes; performed only low-intensity continuous aerobic training (50–70% VO2max); no previous strength or plyometric training experience
Machado et al. 2013	Recreational, endurance-trained	Regional and local level; minimum of 2 years of training experience; training volume of at least 20km/week; the 10-km running times of the participants were between 35 and 60 min; pace between 10 and 17km/h
Daniela et al, 2012	Recreational	Covered 301 km at an average time of 37 hours during the 7 days
Schmid et al, 2012	Athletes	Not presented
Landman et al, 2012	Runners	Average training of 89 km/week.
Knechtle et al, 2011	Athletes	Not presented
Knechtle et al, 2011	Athletes	Not presented
Knechtle et al, 2011	Ultrarunners	Not presented
Knechtle et al, 2010	Recreational and nonprofessional athletes	Not presented

Knechtle et al, 2010	Runners	Not presented
Knechtle et al, 2010	Athletes	Not presented
Knechtle et al, 2009	Athletes	Not presented
Rossuello et al, 2009	Master athletes	Not presented
Manzi et al, 2009	Recreational	Training experience of 5–6 years; absence of clinical signs or symptoms of infection, cardiovascular disease, or metabolic disorders; minimum training distance of 50 km/week
Knechtle et al, 2008	Ultra-endurance runners	Mean training duration of 14.8 (5.5) hours per week; average experience of 10 ultra-endurance races of >24 h
Kilding et al, 2006	Moderately well-trained	Not presented
Nummela et al, 2006	Well-trained	Able to complete 10 km in under 38 min
Sinnett et al, 2001	Recreational	10-km run times ranging from 32.6 to 56.4 minutes, 27 subjects were in training for an upcoming marathon, training volume of 29.7 (15.5) miles/week; trained 5±4.5 days per week for a minimum of 6 months before data collection, 19 of the subjects were involved in weight-training program along with distance training, and 12 of the 36 subjects incorporated some type of speed work into their training regimen, ranging from all-out sprint training to hill running; a total of 27 runners had previously completed a marathon with run times ranging from 3 hours 20 minutes to 4 hours 23 minutes
Wiswell et al, 2000	Master	Not presented
Paavolainen et al, 1999	Well-trained runners	Complete 10 km in under 38 min
Masters & Ogles, 1998	Runners	Not presented
Florence & Weir, 1997	Subjects	Involved in running for a mean of 7.8 (5.2) years; completed at least one marathon
Takeshima & Tanaka, 1995	Competitive runners	Training means 5.4 (1.2) days/week; competitive distance or marathon events for an average of 13.1(7.3) years before the tests; covering 54.5(21.2) km/week and 60.7 (21.2) min day
Ramsbottom et al, 1989	Recreational	Not presented
Marti et al, 1988	Joggers	Not presented

Supplementary Table S2. Summary of the main findings

Authors	Sample characteristics	Performance	Parameters (r; b)
Lempke et al (2026)	♂ 422 ♀ 495	Race time	↑ Running >10 h/week (12–4 and 4–0 months pre-race), more running sessions/week, more quality sessions/week, greater weekly running distance, cross-training sessions/week (4–0 months pre-race), combined increase in running + cross-training sessions (4–0 months pre-race). ↓ Decrease in weekly running sessions (4–0 months pre-race). ↔ Running experience.
Gutiérrez et al (2025)	♂ 871 ♀ 76	Total time	↑ WTn–TT
Knechtle et al (2025)	♂ 732,748 ♀ 125,796	Running speed (km/h)	↑ male, age 35–44, track, road. ↓ Trail, mountain.
Knechtle et al (2025)	♂ ♀ 16,233 race records	Running speed (km/h)	↑ Track races, flat elevation, age ≈45–49 years, athlete countries, event countries (Japan, France, Great Britain, Netherlands, Egypt). ↓ Hilly elevation, older ages. ↔ sex
Turnwald et al (2025)	♂ 69,725 ♀ 20,481	Race speed (km/h)	↑ Age groups 20–24, 25–29, 30–34, 35–39; male; athlete countries (Slovenia, New Zealand, Bulgaria), event countries (New Zealand, Croatia, Serbia). ↓ Age group 40–44
Knechtle et (2014)	♂ 147 (40.2 ± 10.1 years)<br>♀ 83 (38.3 ± 9.2 years)	HH:MM:SS	↑ %BF. ↓ Running speed in training.
Alves et al (2024)	♂ 13 (37,3 ±9,4 years)	HH:MM:SS/ Running pace	HH:MM:SS: ↑ %G (sum DC), ↑ fat mass; ↔ BMI, lean mass, fat-free mass.<br>Running pace: ↑ %G, ↑ fat mass; ↔ BMI, sum DC, residual mass, lean mass, fat-free mass.
Inamura et al, (2024)	♂ 18 men ♀ 4	Running speed	Overall runners: ↔ energy intake, carbohydrate intake<br>Lower-performance group: ↑ energy intake, carbohydrate intake<br>Higher-performance group: ↑ carbohydrate intake<br>All finishers: ↑ carbohydrate intake<br>DNF group: ↔ energy intake, ↔ carbohydrate intake
Knechtle et al (2024)		Running speed (km/h)	↑ Athlete country, age, running surface asphalt, ↓ female, running surface dirt path. ↔ running surface concrete, event country

Martín et al (2024)	♂ 13 (39.5 ± 8.5 years; 57.34 ± 5.03 ml - kg -min VO2MAX)	HH:MM:SS	↓ VVO2MAX. ↑ Arousal, isolation
Thuany et al (2023)	♂♀ 1097	Running pace (s)	Women 5km (↑ BMI. ↓ Training volume. ↔ Age, training frequency, race event, influence to run, family runners.); 10km (↑BMI, influence to run. ↓Training volume. ↔ Age, training frequency, race event, family runners.); Half-marathon (↑ BMI. ↓ Training volume, race event. ↔ Age, training frequency, influence to run, family runners); Marathon (↔ Age, training frequency, training volume, influence to run, family runners. ↑BMI); Men 5km (↑BMI. ↓ Training frequency, training volume. ↔ Age, race event, influence to run, family runners); 10km (↑ Age, BMI. ↓ Training frequency, training volume. ↔ Race event, influence to run, family runners); Half-marathon (↑ Age, BMI. ↓ Training volume. ↔ Training frequency, race event, influence to run, family runners); Marathon (↑BMI. ↓ Training volume, family runners. ↔ Age, training frequency, race event, influence to running).
Nikolaidis et al (2023)	♂ 134 (44.2±8.7 years)	HH:MM:SS	↑ BMI, weekly training volume. ↓VO2max.
Coquart et al (2023)	♂ 56 (48.7 ± 8.8 years)	HH:MM:SS	↑ Perfmarathon, wind speed. ↓ Prmarathon.
Lerebourg et al (2023)	♂♀ 820	HH:MM:SS	↑10km race time, sex, age, BMI .
Siqueira et al (2022)	♂ 20 (38.61 ± 6.36 years)	HH:MM:SS	↓ Post-race plasma levels of IL-6. ↑IL-10 levels.
Ueno et al (2021)	♂ 21 (19.9 ± 1.1 years)	HH:MM:SS	↑ %CT. ↓ CT, StepF, and SL normalized to BH, %SL.
Thuany et al (2021)	♀ 64 Young adults ♀ 177 Adults ♀ 116 Early middle age ♀ 31 Older adults	Running pace (s)	Young adults (↔ BMI, SES, training frequency, training volume, practice time, running club, running event, HDI, athletics events, athletics track, and female homicides); Adults (↑ BMI. ↓ Practice time, training frequency. ↔ SES, training volume, running club, running event, HDI, athletics events, athletics track, and female homicides). Early middle age (↑ Training volume, HDI. ↓BMI. ↔ SES, training frequency, practice time, running club, running event, athletics events, athletics track, and female homicides). Older adults (↑ Practice time. ↓ SES, running events, female homicides. ↔ BMI, training frequency, training volume, running club, HDI, athletics track).
Thuany et al (2021)	♂ 711 ♀ 440 (37.9± 9.4 years)	Running pace (s)	↑ Age, BMI, SES, natural environment, training frequency, training volume, athletic events. ↓ Sex, training frequency, and training volume. ↔ Place of residence, physical structure, HDI, and woman homicides.

Del Rosso et al (2021)	♂ 27 (26.4 ± 6.5 years)	HH:MM:SS	↑ CR, Half Squat-PV, Δ3-Pre CMJPVE, LSJPF. ↓ SMAX, HRmax 10-km.
Martinez-Navarro et al (2021)	♂ 19 (40 ± 5 years) ♀ 13 (42 ± 6 years)	HH:MM:SS	↑ CoefV, SedTimesABS. ↓ IP.
Knechtle et al (2021)	♂ 711,136 ♀ 158,338	HH:MM:SS	↑ Age, sunshine duration, and maximum temperature.
Coates et al (2020)	♂♀ 51 (18-60 years)	HH:MM:SS	Men 50km (↑MAP. ↓ Peak velocity); Women 50km (↑ Body mass. ↓ Peak velocity); 80km (↓ Peak velocity).
Nikolaidis & Knechtle (2020)	♂ 135 (44.2 ± 8.8 years)	HH:MM:SS	↑F0, Pmax. ↔ v0, rPmax.
Matos et al (2020)	♂ 25 (36.23 ± 8.30 years)	Pace	Pace in ST (↓ AcwrTT, tmRPE. ↔ Elac, MAS, alTD, alTT, alsRPE, acwrRPE, tmTD, tmT, tsTD, tsTT, tsRPE); Pace in LT (↑Elac. ↓ AlsRPE, acwrTT. ↔MAS, alTD, alTT, acwrRPE, tmTD, tmTT, tmRPE, tsTD, tsTT, tsRPE); Pace in UT-M (↑ Elac, MAS. ↓ AlsRPE, tsTD, tsTT, tsRPE. ↔ AlTD, alTT, acwrTT, acwrRPE, tmTD, tmTT, tmRPE); Pace in UT-L/XL (↑ Elac. ↔ MAS, alTD, alTT, alsRPE, acwrTT, acwrRPE, tmTD, tmTT, tmRPE, tsTD, tsTT, tsRPE).
Alvero-Cruz et al (2019)	♂ 11 (36 ± 6.5 years)	HH:MM:SS	↑Fat mass. ↓VO2max.
Alvero-Cruz et al (2019)	♂ 23(41.6 ± 7.4 years)	HH:MM:SS	Cooper test (↓Distance covered Cooper test); Treadmill test (↑ Weight. ↓ vVO2max).
Ueno et al (2019)	♂ 42 (20.0 ± 1.0 years)	Pesonal best	↑ Tibia, femur relative leg bone length, femur/tibia ratio of leg bone length. ↓ Tibia relative leg bone length. ↔ Absolute femur and tibia length, absolute total leg bone length femur.
Scheer et al (2019)	♂ 25 (31.23 ± 5.12 years)	HH:MM:SS	↓ LT4.

Rubaltelli (2018)	♂♀ 237(44 ± 8 years)	HH:MM:SS	↑ The desired time reported by runners. ↓ Trait EI.
Ueno et al (2018)	♂ 45 (20.4 ± 2.4 years)	Personal best	↓Relative total length of the forefoot bones (big toe). ↔ Relative total length of the forefoot bones (second toe).
Ueno et al (2018)	♂ 48 (20 ± 1 years)	Personal best	↑Energy cost during submaximal running at 14 km, 16 km, and 18 km. ↓ Passive plantar flexor stiffness.
Fornasiero et al (2017)	♂♀ 23 (40.2 ± 7.3 years)	HH:MM:SS	↑ age. ↓ PowerMax. ↔ BMI, body fat, VO2max, VO2@VT2, power@VT2, VO2@VT1, power@VT1.
Gomez-Molina et al (2017)	Phase 1: ♂ 48 (31.5 ± 7.2 years) Phase 2: ♂ 30 (34.2 ± 6.8 years)	HH:MM:SS	↑ BMI, sum of six skinfolds. ↓ Weekly training volume, running experience, peak speed, RCT speed, RCT speed rate, RCT step length, maximal step length, running experience.
Clemente-Suarez & Nikolaidis (2017)	♂ 52 (38.3 ± 10.1 years)	HH:MM:SS	↓ Years of practicing sports, minutes of daily training. ↔ Years practicing athletics, days of training per week, hours of training per week.
Balducci et al (2017)	♂ 26 (41.7 ± 9.5 years)	HH:MM:SS	↓MAS, fraction of MAS.
Adams et al (2017)	♂ 16 (40 ± 12 years)	HH:MM:SS	↑ %BML, faster average pace, %OFF. ↓ TGI≥ 40C. ↔ Pre-race TGI, post-race TGI.
Forsyth et al (2017)	♂ 18 (49.1±7.0 years) ♀ 18 (45.5±7.0 years)	HH:MM:SS	↔ Tlac-log, Tlac-4mM, Tlac-vis.
Radosavljevic et al (2016)	♂ 11 (16 to 49 years).	HH:MM:SS	↓ Post-run salivary testosterone concentrations. ↔ Salivary cortisol concentrations, before the run salivary testosterone concentrations.
Valentino et al (2016)	♂ 23 ♀ 7	HH:MM:SS	↔ Mean core temperature over the course of the race.
Dellagrana et al (2015)	♂ 23 (18.0 ± 0.9 years)	HH:MM:SS	↓ Vvt, RE11.2, FFM
Kubo et al (2015)	♂ 64 (20.4± 1.2 years)	HH:MM:SS	Knee extensors (↓ Stiffness of tendon structures. ↔ MVC, muscle thickness, maximal elongation of tendon structures, thickness of tendon); Plantar flexors (↓

			Stiffness of tendon structures. ↔ MVC, muscle thickness, maximal elongation of tendon structures, thickness of tendon).
Bertuzzi et al (2014)	♂ 28 (36 ± 8 years)	HH:MM:SS	↑ RPESTART, PTS, Vo2max, 1RM, RCP.
Machado et al (2013)	♂ 27 (40.5 ± 12.1 years)	HH:MM:SS	↑ Vpeak-P during P3 min.
Schmid et al (2012)	♀ 42 (38.5 ± 8.9 years)	HH:MM:SS	↑Mid-axilla skin-fold thickness. ↓ Mean speed of training sessions. ↔ Body mass, BMI, %BF, pectoral skin-fold thickness, triceps skin-fold thickness, subscapular skin-fold thickness, suprailiac skin-fold thickness, abdominal skin-fold thickness, suprailiac skin-fold, medial skin-fold thickness.
Daniela et al (2012)	♂ 10 (49.8 ± 7.4 years) ♀ 2 (48.5 ± 2.5 years)	HH:MM:SS	↑ Tactics in the race. ↔ Years as an active runner, average yearly training volume, the number of completed similar races, resting heart rate, pre-race and post-race body temperature, or values of hematocrit.
Landman et al (2012)	♂♀ 101 (24-61 years)	HH:MM:SS	↔Weight loss, narrow pulse pressure.
Knechtle et al (2011)	♂ 42 (38.5 ± 8.9 years)	HH:MM:SS	↑ Mid-axilla skin-fold. ↓ Mean speed of the training sessions. ↔ Body mass, BMI, %BF, pectoral skin-fold, triceps skin-fold, subscapular skin-fold, abdominal skin-fold, suprailiac skin-fold, medial calf skin-fold.
Knechtle et al (2011)	♂ 63 (46.9 ± 10.3 years)	Kilometers	↑ Longest training session before the 24-h run. ↓ Personal best time in a marathon. ↔ Body mass, sum of 9 skinfolds, %BF, weekly kilometers ran, sum of upper body skinfolds.
Knechtle et al (2010)	♂ 42 (38.5 ± 1.4 years)	HH:MM:SS	↑ Body mass, pectoral skin-fold thickness. ↓ Means peed of training sessions. ↔ BMI, %BF, mid-axilla skin-fold thickness, abdominal skin-fold thickness, suprailic skin-fold thickness, medial calf skin-fold thickness, personal best time in half-marathon.
Knechtle et al (2010)	♂ 52 (39.0 ± 1.4 years) ♀ 15 (35.8 ± 8.8 years)	HH:MM:SS	↑ Body height. ↓ Sex, body mass, BMI, average speed of the training sessions. ↔ %BF, triceps skin-fold, front thigh skin-fold, medial calf-skinf-fold, sum of 8 skinf-folds.

Knechtle et al (2010)	♂ 25 (45.0 years)	HH:MM:SS	↑ Average weekly kilometres in running, average weekly hours in running, personal best time in a marathon. ↓ Average speed in running during training. ↔ Age, body height, body mass, circumferences of limbs (upper arm, thigh, calf), skin-fold thicknesses (pectoral, axillar, triceps, subescapular, abdominal, suprailiacal, thigh, calf), sum of upper-body skinfolds and lower-body skin-folds, sum of 8 skin-folds, BMI, %BF, years as active runner, average weekly hours in running.
Knechtle et al (2010)	♂ 169 (46.5 ± 10.2 years)	HH:MM:SS	↑ Personal best marathon time, age. ↓ Training speed, training volume, %BF.
Knechtle et al (2009)	♂ 15 (46.7 ± 5.8 years)	Kilometers	↑ Personal best time in a marathon, personal best in a 24-hour run. ↔ Age, body height, body mass, length of leg, C upper arm, C thigh, C calf, SF pectoral, SF axillar, SF triceps, SF subscapular, SF abdominal, SF suprailiacal, SF thigh, SF calf, BMI, SM, %BF, training volume, years of competitive running, number of finished marathons, number of finished 24-h runs.
Rossuello et al (2009)	♂ 51 (53.6 ± 8.4 years)	Personal best	↑VO2max, power at LT.
Manzi et al (2009)	♂ 8 (39.9 ± 6.5 years)	HH:MM:SS	5Km (↓ TRIMPi. ↔ TRIMPBan); 10km (↓ TRIMPi. ↔ TRIMPBan).
Knechtle et al (2008)	♂ 19 (46.2 ± 9.6 years)	HH:MM:SS	↑ Arm circumference. ↔ Body height, body mass, average skin-fold thickness, limb circumferences of thigh and calf, leg length, BMI, %BF, and %SM.
Kilding et al (2006)	♂ 36 (23.5 + 5.1 years)	Speed (km/h)	↑VO2max, VT, velocity at VO2max. ↔running economy.
Nummela et al (2006)	♂ 18 (23.4±6.6 years)	Meters by seconds	↑VO2max, MART.
Sinnett et al (2001)	♂ 20 (27.9 ± 5.7 years) ♀ 16 (26.8 ±5.1 years)	HH:MM:SS	Total sample (↑ 300-m Sprint time. ↓ Plyometric leap distance); Men (↓ Plyometric leap distance, 300-m Sprint time); Women (↑ Body weight. ↓ Plyometric leap distance, 300-m Sprint time).
Wiswell et al (2000)	♂ 111 ♀ 57 (40 years or older)	Personal best	Men (↑ Age, km.wk-1, and VO2max. ↔ absolute VO2); Women (↑km.wk-1, VO2max, LT in both 5km and 10km).
Paavolainen et al (1999)	♂ 18 (23.4±6.6 years)	Meters by seconds	↑ VO2max, MART.
Masters & Ogles (1998)	♂♀ 127 (38.5 ± 9.77 years)	Speed (km/h)	↔ Dissociation training.
Florence & Weir (1997)	♂♀ 12 (29 ± 4 years)	HH:MM:SS	↑ Thvent. ↓ CV.

Takeshima & Tanaka (1995)	♂ 51 (57.3 ± 8.9 years)	Speed (km/h)	5km (↑Vo2@LT, average running duration per workout. ↓ Age); 10 km (↑ Vo2@LT, average running duration per workout. ↓Age); Vm (↑Vo2@LT, average running duration per workout. ↓ Age).
Ramsbottom et al (1989)	♂ 18 (25.6 + 6.4 years)<br>♀13 (23.7 ±5.9 years)	HH:MM:SS	Men (↓ Maximal oxygen uptake. ↔ Muscle fibre composition); Women (↓ Maximal oxygen uptake. ↔ Muscle fibre composition).
Marti et al (1988)	♂ 4358	HH:MM:SS	↑ Smoking, BMI, and alcohol consumption. ↓ Years of regular running and training frequency.

Legend: Body mass index (BMI); percentage skeletal muscle mass (%SM); percentage of body fat (%BF), body mass index (BMI), circumference C, skin-fold thickness (SF), skeletal muscle mass (SM); velocity at ventilatory threshold (VVT), running economy at velocity of 11.2 km/h (RE11.2), body fat-free mass (FFM); maximal oxygen consumption (VO2max), oxygen consumption at ventilatory thresholds (VO2@VTs), maximal power output (PowerMax), power output at ventilatory thresholds (Power@VTs); contact time (CT); flight time (FT); step lenght (SL); step frequency (StepF); respiratory compensation threshold (RCT); socioeconomic level (SES); Human development index (HDI); Mean Arterial Pressure (MAP); Race time in marathon (Perfmarathon), personal record during the marathon (PRmarathon); Maximal Aerobic Speed (MAS); muscle force (F0); muscle velocity (v0); absolute maximal power (Pmax); relative power (rPmax); maximal anaerobic running test (MART); maximal treadmill speed (SMAX), gas exchange threshold (GET), anaerobic threshold (VT1), threshold at the respiratory compensation point (VT2); peak velocity during the half squat exercise with the load of 1 $m{\cdot}s^{-1}$ (Half Squat-PV); difference in the CMJ peak eccentric velocity between Pre-10-km and Post 3 min (Δ3-Pre CMJ_{PVE}); maximal heart rate during the 10-km (HRmax 10-km); peak force during the loaded squat jump with the load of 1 $m{\cdot}s^{-1}$ (LSJPF); inflammatory markers (IL-6; IL10); Coefficient of variation (CoefV), index of Pacing (IP), sedentary time (SedTimeabs); speed at VO2max (vVO2max); mean running velocity (Vm); ventilatory threshold (VT); Critical velocity (CV); Cost of running (CR); gastrointestinal temperature (TGI), percent body mass loss (%BML); Aerobic lactate threshold (LTAET), lactate threshold at 4 mmol/l (LT4), individual lactate threshold (IAT); maximal anaerobic running test (MART); Squat jump (SJ), counter movement jump (CMJ), maximal estimated load (MEL); Lactate threshold (LT); Maximal voluntary contraction (MVC); Elevation accumulated (ELac), Maximal aerobic speed (MAS), acute load total distance (alTD), acute load total time (alTT), acute load session-RPE (alsRPE), acute:chronic workload ratio total distance (acwrTD); acute:chronic workload ratio total time (acwrTT), acute:chronic workload ratio RPE (acwrRPE), training monotony total distance (tmTD), training monotony total time (tmTT), training monotony RPE (tmRPE), training strain total distance (tsTD), training strain total time (tsTT), training strain RPE (tsRPE); training impulses (TRIMP), individualized TRIMP (TRIMPi), TRIMP method as proposed by Banister ($TRIMP_{Ban}$); rating of perceived exertion measured during first 400 m (RPESTART), maximum dynamic strength (1RM), respiratory compensation point (RCP), Peak treadmill speed (PTS); The log-log (Tlac-log), The 4-mmol/L method of determining lactate threshold (Tlac-4mM), The visual method of determining lactate threshold (Tlac-vis); Emotional intelligence (EI); ventilatory threshold (Thvent); ↑ correlation/association positive and significant based on the study results; ↓ correlation/association negative and significant based on the study results; ↔ correlation/association non-significant based on the study results. If data were stratified by sex, but the analysis was also estimated for the total sample, we reported the total sample.

Supplementary Table S3. Proposed minimum reporting set for runners

**Domain**	**Variables to report**	**Information**
Participant Identification	Age	Mean ± Standard Deviation or range
	Sex	Male, Female, Other
	Anthropometrics	Height, Weight, Body Mass Index, % Body Fat (when available)
Training History	Years of running experience	Total years consistently running
	Weekly training frequency	Days per week (e.g., 3–6 days/week)
	Average session duration	Minutes per session (e.g., 45–90 min)
	Weekly training volume	Kilometers per week (e.g., 30–100 km/week)
	Training intensity	% time in training zones, RPE, type of training (continuous, interval, fartlek, strength)
	Cross-training / complementary training	Cycling, swimming, resistance training, stretching, physiotherapy
Specialty / Competition	Primary race distance	5 km, 10 km, half-marathon, marathon, ultramarathon
	Race characteristics	Road, trail, cross-country, sky run
	Runner classification	Recreational, Amateur, Trained, Competitive, Master (with criteria used for classification)
	Race experience	Number of races completed per distance, participation in regional/national/international events
	Recent performance	Personal best or recent race times in main distance, trend of performance over past 12 months
	Race characteristics	Road, trail, cross-country, ultramarathon, track
Eligibility / Health Criteria	Injury or illness status	Free from injury/illness at data collection
	Injury history	Type, severity, and time off training in last 6–12 months

	Specific exclusion criteria	Minimum weekly volume, training experience, inability to complete recent race, cardiovascular or musculoskeletal conditions
**Objectives / Goals**	Running purpose	Health, recreation, competition, performance
